# Hungarian PROMIS-29+2: psychometric properties and population reference values

**DOI:** 10.1007/s11136-023-03364-7

**Published:** 2023-02-15

**Authors:** Balázs Jenei, Alex Bató, Ariel Z. Mitev, Valentin Brodszky, Fanni Rencz

**Affiliations:** 1 Department of Health Policy, Corvinus University of Budapest, 8 Fővám Tér, Budapest, 1093 Hungary; 2 Károly Rácz Doctoral School of Clinical Medicine, Semmelweis University, Budapest, Hungary; 3 Institute of Marketing and Communication Sciences, Corvinus University of Budapest, Budapest, Hungary

**Keywords:** PROMIS-29+2, Psychometrics, Validity, Item response theory, Population norm

## Abstract

**Objectives:**

This study aims to assess psychometric properties of the Hungarian PROMIS-29+2 profile measure and provide general population reference values for Hungary.

**Methods:**

An adult general population sample (*n* = 1700) completed PROMIS-29+2 v2.1 in an online survey. The following psychometric properties were assessed: floor and ceiling effect, convergent validity with SF-36v1 domains, internal consistency (McDonald’s omega), unidimensionality, local independence, monotonicity, graded response model (GRM) fit and differential item functioning (DIF). Age- and gender-specific reference values were established using the US item calibrations.

**Results:**

Depending on scale orientation, high floor or ceiling effects were observed for all domains (25.2–60.7%) except for sleep disturbance. McDonald’s omega for domains ranged from 0.87–0.97. Unidimensionality, local independence and monotonicity were supported and the GRM adequately fitted for all but one domains. The sleep disturbance domain demonstrated item misfit, response level disordering and low discrimination ability, particularly for item Sleep116 (‘refreshing sleep’). Strong correlations were observed between PROMIS-29+2 and corresponding SF-36 domains (r_s=_│0.60│ to │0.78│). No DIF was detected for most sociodemographic characteristics. Problems with physical function, pain interference and social roles tended to increase, whereas problems with anxiety, depression, fatigue and cognitive function declined with age (*p* < 0.01). In all domains except for cognitive function, more health problems occurred in females than in males (*p* < 0.001).

**Conclusion:**

The Hungarian PROMIS-29+2 shows satisfactory psychometric properties; however, the sleep disturbance domain substantially underperforms that requires further attention. Population reference values were generated that facilitate the interpretation of health outcomes in various patient populations.

**Supplementary Information:**

The online version contains supplementary material available at 10.1007/s11136-023-03364-7.

## Introduction

In recent years, clinicians, health service providers, researchers, the pharmaceutical industry, reimbursement agencies and health policymakers have been increasingly recognizing the importance of measuring health-related-quality of life (HRQoL) [1, 2]. Some HRQoL instruments are referred to as ‘generic measures’ that describe health in a general way allowing the assessment of HRQoL and changes in HRQoL across a range of disease areas and patient populations, including members of the general public and patient groups. Such measures include the 36-Item Short Form Survey (SF-36), EQ-5D and Assessment of Quality of Life (AQoL) [3, 4]. More recently, the Patient-Reported Outcomes Measurement Information System (PROMIS) adult generic profiles (PROMIS-57, -43 and -29)[5] have been developed that represent a new generation of such measures by relying on item response theory (IRT) calibrated item banks there using a different approach than conventional measures [6].

The PROMIS initiative has so far developed item banks for over 100 key HRQoL domains, such as physical (e.g., pain, physical function, itch, sleep), mental (e.g., anxiety, depression) and social health (e.g., ability to participate in social roles and activities) [7]. Item banks enable computerized adaptive testing (CAT) tools for individual assessment of HRQoL. A major advantage of the three PROMIS generic profile measures is that they are able to produce comparable results to the complete item banks [5]. Although originating from the US, the item banks and the profile measures have been translated to several languages and have increasingly been used in European and Asian countries [8–12]. As standardised HRQoL measures are required to maintain their psychometric performance in different languages, the robustness of measurement properties needs to be confirmed for all language versions.

Among the three PROMIS adult profile measures, PROMIS-29 is the most widely used as a standalone, concise HRQoL measure [13]. By extending it with two items of cognitive function (PROMIS-29+2), it allows the estimation of quality-adjusted life years (QALYs) to assess benefits of treatments in economic analyses [14]. Psychometric performance of PROMIS-29, including validity, reliability and responsiveness, has already been tested in a broad range of health conditions and populations, such as cancer [15, 16], inflammatory bowel diseases [17], chronic kidney disease [18], burn [19], haemophilia [20], musculoskeletal diseases [21–23], systemic lupus erythematosus [24], aortic dissection [25], elderly with multiple chronic conditions [26] and general population [27–30]. Moreover, PROMIS-29 population reference values have also been established in many countries [28, 29] supporting the interpretation of scores by evaluating the relative burden of health conditions compared with reference values. The psychometric performance of the Hungarian PROMIS profile measures has not yet been tested and no reference scores are available for Hungary. This study therefore aims to (1) assess psychometric properties of the Hungarian PROMIS-29+2 profile measure and (2) provide general population reference values from a large representative sample in Hungary.

## Methods

### Study design and data collection

The study was approved by the Research Ethics Committee of the Corvinus University of Budapest (No. KRH/343/2020). The validation of PROMIS-29+2 formed part of a larger survey on health and well-being of the Hungarian general population [31, 32]. In November 2020, a web-based cross-sectional survey was undertaken in Hungary. We engaged a survey research company to conduct the data collection among members of an online panel. By contract the company provided access to the dataset of those respondents’ responses that had fully completed the questionnaire. Providing access to partially completed questionnaires was not included in the contract. The survey company provided compensation to the respondents in the form of survey points redeemable for rewards. We set ‘soft’ target quotas for age, gender, education, type of settlement and region to achieve a sample that approximates the composition of the Hungarian adult general population. Inclusion criteria were being aged ≥ 18 years and providing informed consent prior to starting the survey.

Respondents completed the official Hungarian-language version PROMIS-29+2 v2.1 [33] as distributed by the PROMIS Health Organization. Other data collected included sociodemographic questions (age, gender, education, employment, marital status, income, household size, type of settlement, region), history of chronic health conditions and the 36-item Short Form Health Survey (SF-36v1). The order of the two instruments was fixed, respondents first completed the PROMIS-29+2 followed by the SF-36. There were no missing values in the data as we made it mandatory to respond to all questions in the online survey.

### *PROMIS-29*+*2*

PROMIS-29+2 v2.1 [33] was included in our survey that consists of PROMIS-29 and two items from Cognitive Function-Abilities v2.0 [34]. The PROMIS-29 profile comprises of 29 items relating to the following seven HRQoL domains [physical function, anxiety, depression, fatigue, sleep disturbance, ability to participate in social roles and activities (hereafter social roles) and pain interference] and an 11-point pain intensity numeric rating scale [5]. The Cognitive Function-Abilities items are measures of an eighth, cognitive function domain. Each PROMIS-29 domain has four five-level items. The five-point response scale varies across difficulty (i.e., ‘without any difficulty’ to ‘unable to do’), frequency (‘never’ to ‘always’), severity (‘not at all’ to ‘very much’) and global rating (‘very poor’ to ‘very good’) format scales. The recall period is unspecified for physical function and social roles; all other domains refer to the past seven days. A total raw score ranging from 4 to 20 (2–10 for cognitive function) may be computed for each domain by adding up the responses on each item of the domain. The US item calibrations were used to derive T-scores from raw domain scores, where a mean T-score of 50 with a SD of ten represents the US general population [7]. The only exception is the sleep disturbance domain, where a mixed general population and clinical sample was used for the calibration of T-scores with above-average sleep disturbance [35]. For scales of function (i.e., physical function, social roles and cognitive function) a higher score corresponds to a better HRQoL and for symptoms (i.e., anxiety, depression, fatigue, sleep disturbance and pain interference) a higher score corresponds to worse HRQoL [36].

### *36-item short form survey (SF-36)*

SF-36 is one of the most extensively used and validated generic HRQoL instruments [37]. It assesses respondents’ HRQoL in 36 items covering eight domains with a four-week recall period: physical functioning (ten items), role limitations due to physical health problems (four items), bodily pain (two items), general health (five items), vitality (four items), social functioning (two items), role limitations due to emotional problems (three items) and mental health (five items). One item (2nd), which asks about health change, is not included in the scale or summary scores. Scores for items on each of the eight scales are summed up to give scale scores that are linearly transformed onto a 0–100 scale. Note that scores are not comparable across domains.

### Psychometric analyses

Data analysis was carried out with R version 4.1.1 (Vienna, Austria). We followed classical test theory and IRT methods previously used in testing psychometric properties of PROMIS item banks and profile measures [6, 20, 21, 27, 38, 39]. For the analyses, we considered PROMIS-29 as the core measure and we tested measurement properties of the additional cognitive function domain separately, wherever possible. Psychometric analyses were performed on the unweighted sample; however, for estimating population reference values, the sample was weighted for age group and gender. All the statistical tests were two-sided, and *p* < 0.05 was considered statistically significant.

#### Floor and ceiling effect

Floor (proportion of responses at the lowest score) and ceiling (proportion of responses at the highest score) were computed for the eight PROMIS-29+2 domains. If > 15% of respondents scored the lowest or highest response level, we considered ceiling or floor effect to be present [40, 41].

#### Reliability analyses

Internal consistency reliability was assessed by computing Cronbach’s alpha and McDonald’s omega (total) for each domain (*‘psych’* package [42]). For Cronbach’s alpha, a value > 0.70, while for McDonald’s omega total > 0.90 was considered as a sign of adequate internal consistency [43].

#### Item response theory assumptions

In accordance with previous PROMIS validation studies [6, 27, 30], the seven domains of PROMIS-29 were separately analysed with graded response models (GRM). Before modelling, the following three statistical assumptions were tested: unidimensionality, local independence and monotonicity. Unidimensionality was assessed using an exploratory bifactor model (*‘psych’* package [42]) that allowed to extract explained common variance (ECV) and McDonald’s omega (hierarchical) values. The following cut-off values were used: ECV > 0.60 and omega > 0.70 [44]. IRT-based standardized Chen and Thissen’s index (χ^2^) was used to detect local dependence (*‘mirt’* package [45]). A χ^2^ of > 0.3 implied possible local dependence and > 1 definite local dependence [46]. Any violations of local dependence were considered negligible if the ECV was ≥ 0.90 [46–49]. Monotonicity was tested by examining the graphs of item mean scores conditional on the total raw scale score minus the item score [6].

#### Item response theory analyses

After confirming the IRT assumptions, we fitted a GRM (*‘mirt’* package [45]). We examined each item’s discrimination (i.e., item slope, a) and item thresholds (i.e., item difficulty, b). Model fit was assessed by root mean square error of approximation (RMSEA), Standardized Root Mean Square Residual (SRMR), Comparative Fit Index (CFI) and Tucker–Lewis Index (TLI), and was considered acceptable if CFI > 0.95, TLI > 0.95, RMSEA < 0.06 and SRMR < 0.08 [50]. Item fit was assessed by computing the differences between observed and expected responses under the GRM using S-χ^2^ statistic, where a *p*-value < 0.001 was considered indicative of item misfit [51]. Item characteristic curves (ICCs) were generated using GRM.

#### Differential item functioning

To assess differential item functioning (DIF), a series of ordinal logistic regressions were fitted (*‘lordif’* package [52]). In the first step, we performed an ordinal logistic regression without any anchor. The χ^2^ criterion was assessed looking for potential items with DIF. Once DIF was detected, we moved to the second step, where items within a domain that did not show any DIF were used as already-purified anchors. In this second step, three ordinal logistic regression models were estimated to compare the overall, uniform and non-uniform DIF for each item. Uniform DIF occurs when there is a constant systematic difference in item response between subgroups of respondents across the entire continuum of the latent trait, whereas non-uniform DIF occurs when the differences between groups vary across the continuum of the latent trait. Uniform, non-uniform and overall DIF were examined by comparing model 1 vs. model 2, model 2 vs. model 3, model 1 vs. model 3, respectively. Items were flagged for DIF when the McFadden’s pseudo *R*^2^ change was > 0.02 [33]. Test characteristic curves were used to visualize the aggregate impact of DIF on domain scores (i.e., differential test functioning). DIF was evaluated for age (median split at 47 years), gender (male vs. female), education (primary, secondary, university/college), employment (employed, retired, other), place of residence (capital, other town, village), geographical region (Central Hungary, Transdanubia, Great Plain and North), marital status (married or domestic partnership vs. any other) and household net monthly income per person (under or over the median of HUF 126,924 and do not know/want to answer).

#### Convergent validity

Convergent validity of PROMIS-29+2 was assessed against the SF-36v1 questionnaire. We used Spearman’s rank-order correlations to test the association between domains and summary scores of the two measures. Correlation coefficients were interpreted as very weak (< 0.20), weak (0.20–0.39), moderate (0.40–0.59), strong (0.60–0.79) and very strong (≥ 0.80) [53]. We hypothesized at least strong correlations between domains covering a similar construct (e.g., PROMIS physical function and SF-36 physical functioning). Weak or no correlations were assumed between the PROMIS cognitive function and SF-36 domains as this area of HRQoL is missing from the SF-36.

### Population reference values and cross-country comparisons

In estimating population reference values, the sample was weighted for age group and gender to account for small deviations from the reference population in Hungary [54]. To accommodate the effect of weighting on variances, Taylor linearization was used to calculate appropriate standard errors. Mean (SD) dimension and summary T-scores and their 95%CIs were computed by gender and age groups (18–24, 25–34, 35–44, 45–54, 55–64 and 65 + years). Bivariate ordinary least squares regressions were used to test the association between domain T-scores and pain intensity scores with age groups and gender. Weighted domain T-scores were compared to those of the general population in the US, the UK, Germany and France [28].

## Results

### Characteristics of the sample

Overall, 2502 online panel members initiated the survey. Of these, 2079 consented and 379 dropped out during the questionnaire. A total of 1700 respondents finished the survey. The median completion time of PROMIS-29+2 was 2 min 59 s (Q1: 2 min 9 s, Q3: 4 min 8 s). Table 1 shows the sociodemographic and health-related characteristics of the respondents in comparison to the general population in Hungary. The sample was generally representative of the Hungarian general population for age, gender, employment and marital status, type of settlement and geographical region. Secondary educated respondents were underrepresented in the sample. Overall, 47.4% had a self-reported, physician diagnosed health condition. Descriptive statistics of PROMIS-29+2 and SF-36 domain scores are presented in Table 2.

**Table 1:** Characteristics of the study population (*n* = 1700)

Variables	Ref. population^a^	Sample	
	%	*n*	%
Gender			
Female	53.1	957	56.3
Male	46.9	743	43.7
Age (years)			
18–24	10.0	148	8.7
25–34	15.2	293	17.2
35–44	19.5	309	18.2
45–54	16.0	304	17.9
55–64	16.8	296	17.4
65 +	22.5	350	20.6
Highest level of education			
Primary school or less	23.8	468	27.5
Secondary school	55.0	682	40.1
College/university degree	21.2	550	32.4
Settlement			
Capital	17.9	380	22.4
Other town	52.6	820	48.2
Village	29.5	500	29.4
Geographical region			
Central Hungary	30.4	572	33.6
Transdanubia	30.2	493	29
Great Plain and North	39.5	635	37.4
Employment status			
Employed	53.1	865	50.9
Retired	26.1	399	23.5
Disability pensioner	3.1	67	3.9
Student	4.7	74	4.4
Unemployed	3.1	129	7.6
Homemaker/housewife	1.0	99	5.8
Other	n/a	67	3.9
Per capita net monthly household income (HUF)			
0–66,779	n/a	224	13.2
66,780–99,511	n/a	252	14.8
99,512–126,924	n/a	229	13.5
126,925–164,049	n/a	207	12.2
164,050 +	n/a	423	24.9
I do not know/refused to answer	n/a	365	21.5
Marital status			
Married	45.6	718	42.2
Domestic partnership	13.4	360	21.2
Single	18.5	336	19.8
Widowed	11.4	98	5.8
Divorced	11.1	156	9.2
Other	n/a	32	1.9
Self-perceived health status *(SF-36 question 1)*			
Excellent	n/a	139	8.2
Very good	n/a	401	23.6
Good	n/a	682	40.1
Fair	n/a	388	22.8
Poor	n/a	90	5.3
History of chronic illness^b^			
Yes	48.0	805	47.4
No	52.0	724	42.6
Do not know/refused to answer	–	171	10.1

n/a = not available

^a^Hungarian Central Statistical Office: Microcensus 2016

^b^Hungarian Central Statistical Office, Health at a Glance 2019

Percentages may not total 100% due to rounding

**Table 2:** Descriptive statistics of the outcome measures

Measures	Theoretical range	Observed range	Floor effect		Ceiling effect		Mean	SD	Median	Q1–Q3
			n	%	n	%				
PROMIS - 29+2										
Physical functioning T-score	22.5–57	22.5–57	5	0.29	1032	60.71	51.55	7.56	57	45.5–57
Anxiety T-score	40.3–81.6	40.3–81.6	601	35.35	11	0.65	50.84	9.81	51.2	40.3–57.7
Depression T-score	41–79.4	41–79.4	749	44.06	13	0.76	49.94	9.54	49	41–55.7
Fatigue T-score	33.7–75.8	33.7–75.8	429	25.24	22	1.29	46.92	10.42	48.6	33.7–53.1
Sleep disturbance T-score	32–73.3	32–73.3	105	6.18	11	0.65	48.39	8.22	48.4	42.45–54.3
Social roles T-score	27.5–64.2	27.5–64.2	13	0.76	664	39.06	55.45	8.89	55.8	50–64.2
Pain interference T-score	41.6–75.6	41.6–75.6	858	50.47	18	1.06	49.35	8.9	41.6	41.6–55.6
Cognitive function T-score	29.5–61.2	29.5–61.2	98	5.76	620	36.47	52.66	8.73	54.7	50.5–61.2
Pain intensity NRS (0–10)	0–10	0–10	530	31.18	7	0.41	2.49	2.5	2	0–4
SF-36										
Physical functioning	0–100	0–100	14	0.82	637	37.47	81.72	24.15	90	75–100
Role functioning	0–100	0–100	216	12.71	1007	59.24	74.74	36.12	100	50–100
Role emotional	0–100	0–100	207	12.18	1077	63.35	75.98	35.8	100	66.67–100
Vitality	0–100	0–100	11	0.65	113	6.65	62.13	23.88	65	45–80
Mental health	0–100	0–100	6	0.35	164	9.65	69.95	23.12	76	56–88
Social functioning	0–100	0–100	12	0.71	783	46.06	79.83	24.53	87.5	62.5–100
Bodily pain	0–100	0–100	9	0.53	574	33.76	76.16	24.3	80	57.5–100
General health	0–100	0–100	13	0.76	74	4.35	59.52	23.35	60	45–75

For PROMIS-29+2 scales of function (i.e., physical function, social roles and cognitive function) a higher score corresponds to a better HRQoL and for symptoms (i.e., anxiety, depression, fatigue, sleep disturbance and pain interference) a higher score corresponds to worse HRQoL. Higher score on pain intensity NRS indicates worst pain. For all SF-36 domains and summary scores, higher scores indicate better HRQoL. The observed range shows the range of domain scores observed in our sample, while the theoretical range refers to the possible range of the domains/items according to the PROMIS-29+2 and SF-36 instruments

*HRQoL* health-related quality of life, *NRS*  numeric rating scale

### Floor and ceiling effect

Among the eight PROMIS-29+2 domains, the highest floor effects were observed for pain interference (50.5%), followed by depression (44.1%), anxiety (35.4%) and fatigue (25.2%) (Table 2). Floors of the physical function, social roles, sleep and cognitive function domains were well below the threshold (0.3–6.2%). High ceiling effect was observed for physical function (60.7%), social roles (39.1%) and cognitive function (36.5%), while there were no apparent ceiling effects for the other domains (0.4–1.3%).

### Reliability

Cronbach’s alpha and McDonald’s omega total values exceeded the thresholds of 0.70 and 0.90 for all PROMIS-29 domains with the exception of McDonald’s omega total (0.87) for the sleep disturbance domain (Table 3).

**Table 3:** Unidimensionality, IRT model fit and reliability estimates for the domains of the Hungarian PROMIS-29

	Bifactor model (exploratory)		Graded response model				Reliability analyses	
	ECV	McDonald’s ω (hierarchial)	RMSEA	SRMR	CFI	TLI	Cronbach’s α	McDonald’s ω (total)
Physical function	0.80	0.87	0.103	0.025	0.993	0.979	0.91	0.96
Anxiety	0.93	0.91	0.032	0.012	0.999	0.998	0.92	0.94
Depression	0.92	0.92	0.056	0.013	0.998	0.995	0.93	0.94
Fatigue	0.91	0.92	0.126	0.020	0.992	0.975	0.94	0.96
Sleep disturbance	0.68	0.70	0.290	0.089	0.897	0.692	0.81	0.87
Social roles	0.93	0.92	0.035	0.017	0.999	0.998	0.93	0.94
Pain interference	0.94	0.94	0.067	0.010	0.998	0.994	0.96	0.97

*CFI* comparative fit index, *ECV* explained common variance, *IRT* item response theory, *RMSEA* root mean square error of approximation, *SRMR* Standardized Root Mean Square Residual, *TLI*  Tucker–Lewis index

### IRT assumptions

Using bifactor models, the unidimensionality assumption was confirmed for all PROMIS-29 domains. For sleep disturbance, ECV was met (0.68), however, McDonald’s omega hierarchical was exactly at the threshold (0.70) (Table 3). Chen and Thissen’s local dependence indices were below 1 for nearly all item pairs of each domain (Online Resource 1). The exceptions include Sleep109 (‘sleep quality’) vs. Sleep20 (‘problem with sleep’) and PAININ9 (‘pain interfering with day to day activities’) vs. PAININ22 (‘pain interfering with work around the home’). However, for the latter pair, the ECV from the bifactor model was very high (0.94), therefore the local dependence detected can be deemed negligible. In the sleep disturbance domain three item pairs showed a Chen and Thissen’s index of above 0.3 and one pair was above 1. Graph item mean scores conditional on total score minus item score supported the monotonicity assumption for all domains (Online Resource 2).

### IRT analysis

For each of the seven PROMIS-29 domains, almost all three assumptions of IRT analysis were met. Several items misfitted the GRM as indicated by the p-values for the S–χ2 statistics (Table 4). Misfitting items included two items of the anxiety domain [EDANX01 (‘fearful’) and EDANX53 (‘uneasy’)], two items of the depression domain [EDDEP04 (‘worthless’), EDDEP41 (‘hopeless’)], all four items of the sleep disturbance domain and one item of the pain interference domain [PAININ31 (‘pain interfering with social activities’)].

**Table 4:** IRT parameters for the Hungarian PROMIS-29

Item code	Graded response model								
	a	b1	b2	b3	b4	Average b	Index-S-χ2	df	*p*-value
Physical function									
PFA11	3.689	−2.529	−2.014	−1.503	−0.841	−1.72	29.897	18	0.038
PFA21	4.083	−2.423	−1.696	−1.111	−0.422	−1.41	25.186	14	0.033
PFA23	5.856	−2.32	−1.91	−1.466	−1.005	−1.68	17.187	11	0.102
PFA53	4.628	−2.571	−2.038	−1.615	−1.073	−1.82	20.957	14	0.103
Anxiety									
EDANX01	3.926	0.114	0.858	1.608	2.394	1.24	39.719	15	< 0.001
EDANX40	5.348	0.51	1.134	1.721	2.41	1.44	30.455	12	0.002
EDANX41	4.802	0.198	0.898	1.431	2.08	1.15	36.039	15	0.002
EDANX53	3.714	−0.248	0.664	1.349	2.138	0.98	52.853	17	< 0.001
Depression									
EDDEP04	3.819	0.396	0.957	1.592	2.235	1.3	47.713	18	< 0.001
EDDEP06	3.986	0.058	0.701	1.38	2.206	1.09	28.28	16	0.029
EDDEP29	4.191	0.501	0.989	1.604	2.304	1.35	29.28	16	0.022
EDDEP41	6.718	0.294	0.853	1.369	1.941	1.11	69.464	13	< 0.001
Fatigue									
HI7	4.656	−0.422	0.565	1.165	1.984	0.82	35.657	14	0.001
AN3	4.29	−0.142	0.708	1.334	1.963	0.97	30.747	15	0.009
FATEXP41	3.928	−0.233	0.686	1.372	2.134	0.99	15.433	14	0.349
FATEXP40	6.941	−0.401	0.589	1.188	1.873	0.81	17.222	10	0.07
Sleep disturbance									
Sleep109	2.644	−1.145	0.125	1.334	2.3	0.65	110.436	21	< 0.001
Sleep116	1.346	−1.972	−0.244	0.863	1.591	0.06	303.663	27	< 0.001
Sleep20	4.166	−0.199	0.559	1.317	1.955	0.91	95.558	19	< 0.001
Sleep44	2.29	−0.213	0.653	1.333	2.12	0.97	143.605	25	< 0.001
Ability to participate in social roles and activities									
SRPPER11_CaPS	4.413	−2.043	−1.449	−0.766	−0.037	−1.07	18.222	15	0.251
SRPPER18_CaPS	4.948	−2.085	−1.509	−0.93	−0.302	−1.21	16.555	15	0.346
SRPPER23_CaPS	3.556	−2.4	−1.546	−0.872	−0.03	−1.21	16.67	17	0.477
SRPPER46_CaPS	5.4	−1.914	−1.333	−0.783	−0.167	−1.05	29.008	15	0.016
Pain interference									
PAININ9	6.934	0.167	0.914	1.488	2.174	1.19	15.058	8	0.058
PAININ22	8.397	0.252	0.923	1.448	1.952	1.14	10.056	7	0.185
PAININ31	5.904	0.459	1.012	1.499	2.088	1.26	41.178	12	< 0.001
PAININ34	8.177	0.278	0.982	1.474	2.019	1.19	5.093	7	0.649

a = item’s discrimination (item slope), b = item threshold (item difficulty), IRT = item response theory

For all domains but sleep disturbance, the GRM models’ fit indices met the established criteria for SRMR, CFI and TLI. However, out of the seven PROMIS-29 domains, only anxiety, depression and social roles met the RMSEA cut-off value. The sleep disturbance (0.06–0.97) and fatigue (0.81–0.99) domains had the lowest average item difficulty (b), while physical function (1.41–1.82) had the highest in absolute values. The following items produced the highest discriminative ability (a): PAININ22 (‘pain interfering with work around the home’), PAININ34 (‘pain interfering with household chores’), FATEXP40 (‘fatigue on average’) and PAININ9 (‘pain interfering with day to day activities’). Three items of the sleep disturbance domain [Sleep116 (‘refreshing sleep’), Sleep44 (‘difficulty falling asleep’), Sleep109 (‘sleep quality’)] had the lowest item discrimination.

**Fig. 1:**
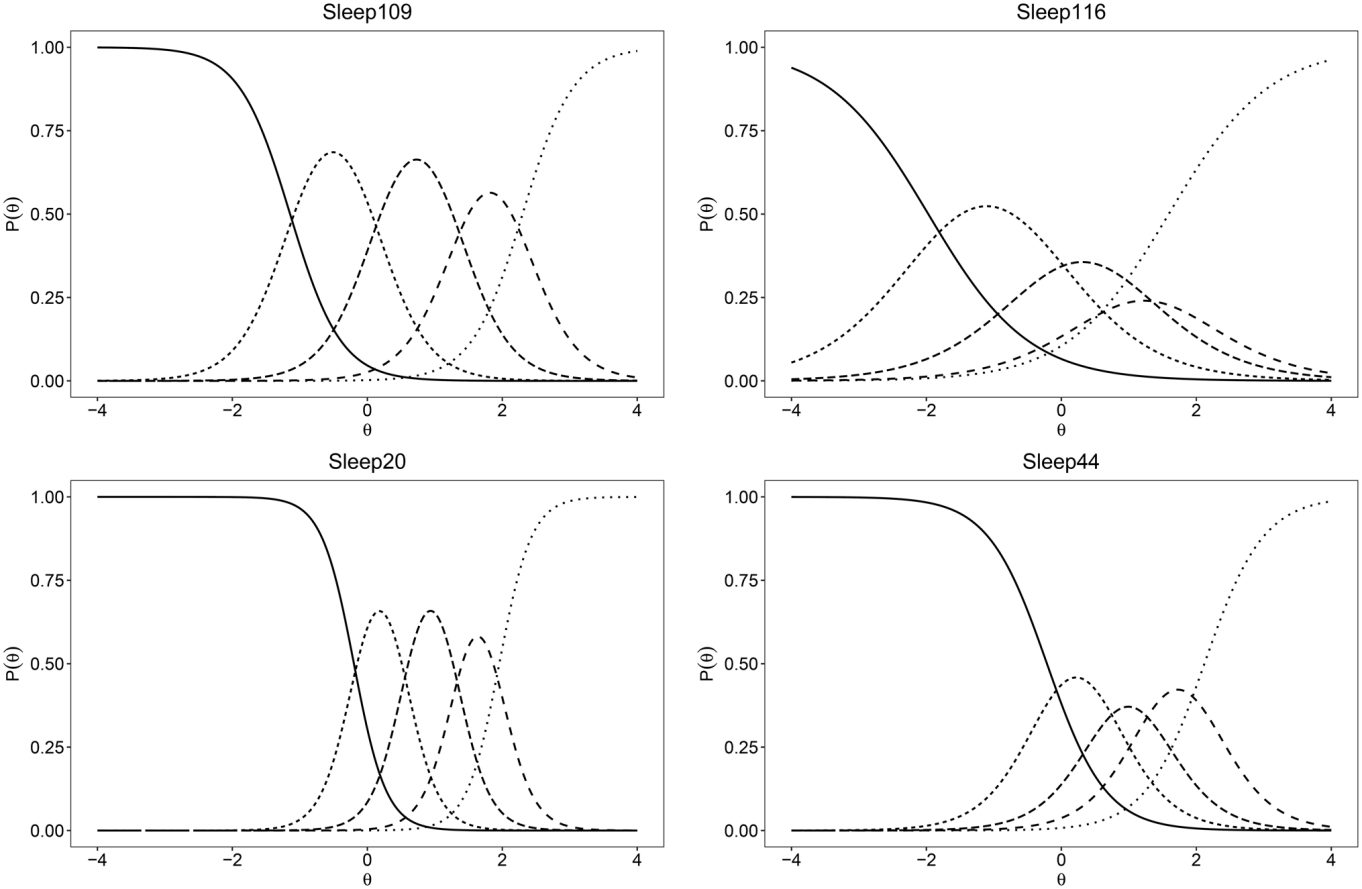
Item characteristic curves for PROMIS-29+2 Sleep disturbance domain

The ICC plots shown in Online Resource 3 indicated that for most items, the five response options were monotonically ordered. The only exception was item Sleep116 (‘refreshing sleep’) (Fig. 1).

### Differential item functioning

No DIF was identified for any of the domains for the following sociodemographic characteristics: gender, education, employment, place of residence, geographical region, marital status and income. However, PFA21 (‘go up and down stairs at a normal pace’) and PFA53 (‘run errands at shop’) of the physical function domain showed uniform DIF for age (McFadden’s pseudo *R*^2^ changes between model 1 and 2: 0.030 and 0.023, respectively). The test characteristic curves for these two items showed a small overall impact of DIF (Online Resource 4).

### Convergent validity

Table 5 presents the results of the convergent validity analyses. In line with our hypotheses, evidence of strong convergence between corresponding PROMIS-29+2 and SF-36 domains were identified. The strongest correlations were observed between PROMIS-29+2 physical function and SF-36 physical function domains (*r*_s_ = 0.78), PROMIS-29+2 fatigue and SF-36 vitality (*r*_s_ = −0.76), PROMIS-29+2 pain interference and SF-36 bodily pain (*r*_s_ = −0.74) and PROMIS-29+2 depression and SF-36 mental health (*r*_s_ = −0.70). The PROMIS-29+2 sleep disturbance domain correlated weakly or moderately with SF-36 domains and showed the strongest association with vitality (*r*_s_ = −0.57). As expected, the PROMIS-29+2 cognitive function domain correlated moderately or weakly with all SF-36 domains (*r*_s_ = 0.18–0.42). The correlations between the domains within the two questionnaires are presented in Online Resources 5 and 6.

**Table 5:** Spearman’s correlation matrix between PROMIS-29+2 and SF-36 domains

PROMIS-29+2	SF-36							
	PF	RP	RE	VT	MH	SF	BP	GH
Physical function	0.78	0.60	0.42	0.38	0.31	0.39	0.56	0.59
Anxiety	−0.27	−0.36	−0.49	−0.60	−0.66	−0.53	−0.39	−0.38
Depression	−0.30	−0.38	−0.50	−0.63	−0.70	−0.58	−0.40	−0.40
Fatigue	−0.40	−0.49	−0.53	−0.76	−0.61	−0.53	−0.53	−0.46
Sleep disturbance	−0.29	−0.32	−0.34	−0.57	−0.54	−0.43	−0.37	−0.39
Ability to participate in social roles and activities	0.55	0.56	0.53	0.61	0.53	0.60	0.54	0.55
Pain interference	−0.64	−0.62	−0.45	−0.48	−0.39	−0.51	−0.74	−0.56
Cognitive function	0.18	0.19	0.26	0.41	0.42	0.39	0.24	0.26
Pain intensity (0–10)	−0.54	−0.53	−0.39	−0.48	−0.40	−0.46	−0.79	−0.54

For PROMIS-29+2 scales of function (i.e., physical function, social roles and cognitive function) a higher score corresponds to a better HRQoL and for symptoms (i.e., anxiety, depression, fatigue, sleep disturbance and pain interference) a higher score corresponds to worse HRQoL

*BP* bodily pain; *GH * general health; *HRQoL* health-related quality of life; *MH* mental health; *PF* physical functioning; *RE* role limitations due to emotional problems; *RP* role limitations due to physical health; *SF* social functioning; *VT* vitality

p < 0.05 for all correlation coefficients

### Population reference values and cross-country comparisons

Mean domain T-scores tended to worsen with age for physical function, pain interference and social roles, whereas improved with age for depression, anxiety, fatigue and cognitive function (*p* < 0.01) (Table 6). The age gradient was not present for sleep disturbance (*p* = 0.155). Self-reported HRQoL problems were generally higher for females in all domains (*p* < 0.001), except for cognitive function (*p* = 0.348). Higher mean pain intensity scores were reported by older and female respondents (*p* < 0.001).

**Table 6:** Population reference values for Hungarian PROMIS-29+2 domain T-scores and pain intensity scale

Age groups (years)	Female					Male					Total				
	n	Mean	SD	95%CI lower	95%CI upper	n	Mean	SD	95%CI lower	95%CI upper	n	Mean	SD	95%CI lower	95%CI upper
Physical function															
18–24	82	53.63	6.90	52.56	54.7	87	55.71	2.66	54.21	57.21	169	54.7	4.88	53.77	55.63
25–34	126	53.05	8.78	52.1	54	133	54.57	4.52	53.42	55.72	259	53.83	6.68	53.08	54.58
35–44	164	52.23	7.34	51.13	53.32	167	54.45	5.24	53.5	55.4	331	53.35	6.38	52.62	54.07
45–54	137	50.3	8.94	48.98	51.61	135	53.66	6.12	52.71	54.62	272	51.97	7.85	51.15	52.78
55–64	154	49.69	8.28	48.41	50.97	132	50.3	8.74	48.87	51.73	286	49.97	8.50	49.02	50.92
65 +	239	46.73	6.23	45.51	47.95	144	49.66	8.80	48.6	50.73	383	47.84	7.46	46.97	48.7
Total	902	50.29	8.20	49.78	50.8	798	52.92	6.62	52.45	53.39	1700	51.53	7.58	51.18	51.87
Anxiety															
18–24	82	54.4	12.08	52.52	56.28	87	49.17	5.25	46.22	52.12	169	51.72	9.11	49.95	53.49
25–34	126	54.33	13.58	52.86	55.79	133	49.78	7.43	47.89	51.68	259	52	10.79	50.79	53.2
35–44	164	52.06	9.60	50.62	53.5	167	49.23	8.84	47.63	50.83	331	50.63	9.33	49.55	51.71
45–54	137	52.77	11.06	51.15	54.4	135	48.74	9.44	47.27	50.21	272	50.77	10.49	49.68	51.87
55–64	154	51.51	10.29	49.92	53.09	132	47.77	8.83	46.33	49.22	286	49.79	9.83	48.7	50.87
65 +	239	51.21	7.68	49.7	52.71	144	47.33	8.86	46.25	48.4	383	49.75	8.66	48.72	50.77
Total	902	52.38	10.36	51.72	53.04	798	48.65	8.55	47.96	49.33	1700	50.63	9.69	50.15	51.1
Depression															
18–24	82	53.43	11.79	51.6	55.27	87	49.52	5.41	46.48	52.56	169	51.43	8.96	49.63	53.23
25–34	126	52.87	13.52	51.41	54.33	133	49.78	7.77	47.8	51.77	259	51.29	10.83	50.05	52.53
35–44	164	51.09	10.34	49.54	52.64	167	49.6	8.51	48.06	51.13	331	50.34	9.44	49.24	51.43
45–54	137	51.27	9.92	49.81	52.72	135	48.16	9.70	46.65	49.67	272	49.73	9.95	48.68	50.78
55–64	154	50.06	9.59	48.57	51.54	132	46.99	8.87	45.54	48.44	286	48.64	9.40	47.6	49.69
65 +	239	49.43	6.80	48.09	50.76	144	46.87	8.71	45.81	47.92	383	48.46	7.83	47.54	49.38
Total	902	50.96	9.93	50.34	51.59	798	48.45	8.64	47.76	49.15	1700	49.79	9.41	49.32	50.25
Fatigue															
18–24	82	50.34	12.42	48.41	52.27	87	46.51	7.02	42.57	50.46	169	48.38	10.41	46.14	50.61
25–34	126	50.53	13.63	49.06	52	133	46.08	8.60	43.89	48.28	259	48.25	11.53	46.91	49.58
35–44	164	48.41	10.30	46.87	49.96	167	46.24	9.77	44.48	48.01	331	47.32	10.11	46.14	48.49
45–54	137	48.4	10.81	46.82	49.99	135	45.11	9.87	43.57	46.64	272	46.77	10.49	45.66	47.87
55–64	154	46.88	10.60	45.24	48.52	132	44.69	10.41	42.99	46.39	286	45.87	10.58	44.69	47.05
65 +	239	46.06	8.18	44.45	47.66	144	43.22	10.40	41.96	44.47	383	44.99	9.38	43.88	46.09
Total	902	48	10.76	47.31	48.68	798	45.25	9.85	44.44	46.06	1700	46.71	10.42	46.18	47.23
Sleep disturbance															
18–24	82	49.25	10.68	47.59	50.91	87	48.63	4.32	46.2	51.06	169	48.93	7.54	47.45	50.42
25–34	126	50.7	10.31	49.58	51.81	133	47.16	6.26	45.56	48.76	259	48.88	8.59	47.9	49.87
35–44	164	48.69	8.24	47.45	49.92	167	48.1	7.00	46.84	49.37	331	48.39	7.62	47.51	49.28
45–54	137	49.7	8.42	48.47	50.94	135	46.13	8.33	44.84	47.43	272	47.93	8.59	47.04	48.83
55–64	154	49.48	8.92	48.1	50.85	132	47.44	8.10	46.11	48.76	286	48.54	8.62	47.57	49.5
65 +	239	49.17	6.98	47.81	50.54	144	45.75	8.40	44.73	46.77	383	47.89	7.95	46.95	48.82
Total	902	49.44	8.71	48.87	50	798	47.13	7.34	46.55	47.72	1700	48.36	8.18	47.95	48.76
Ability to participate in social roles and activities															
18–24	82	56.35	9.75	54.83	57.86	87	57.89	5.09	55.03	60.76	169	57.14	7.75	55.5	58.78
25–34	126	55	11.46	53.76	56.24	133	57.57	6.42	55.93	59.21	259	56.32	9.07	55.28	57.35
35–44	164	55.78	9.06	54.42	57.13	167	57.22	7.68	55.83	58.6	331	56.5	8.39	55.53	57.48
45–54	137	53.88	9.53	52.48	55.28	135	57.56	8.89	56.17	58.94	272	55.71	9.42	54.72	56.69
55–64	154	53.26	9.78	51.75	54.77	132	55.99	9.28	54.47	57.51	286	54.52	9.65	53.44	55.59
65 +	239	52.92	7.09	51.53	54.31	144	55.53	9.76	54.35	56.71	383	53.9	8.36	52.93	54.88
Total	902	54.25	9.33	53.65	54.84	798	56.9	8.17	56.25	57.55	1700	55.49	8.89	55.05	55.93
Pain interference															
18–24	82	47.73	9.14	46.3	49.15	87	45.14	3.52	43.16	47.12	169	46.4	6.45	45.17	47.63
25–34	126	48.76	11.72	47.5	50.03	133	46.54	6.26	44.95	48.14	259	47.63	9.07	46.6	48.65
35–44	164	49.29	9.04	47.93	50.64	167	47.45	7.09	46.17	48.73	331	48.36	8.11	47.43	49.29
45–54	137	52.15	10.49	50.61	53.69	135	47.28	8.02	46.03	48.53	272	49.74	9.66	48.74	50.73
55–64	154	51.18	9.71	49.68	52.68	132	49.14	8.93	47.68	50.6	286	50.24	9.42	49.18	51.29
65 +	239	52.74	7.26	51.32	54.17	144	49.62	10.03	48.4	50.83	383	51.57	8.60	50.57	52.57
Total	902	50.74	9.59	50.14	51.35	798	47.69	7.71	47.11	48.27	1700	49.31	8.85	48.89	49.73
Cognitive function															
18–24	82	49.67	9.87	48.13	51.2	87	48.57	7.10	44.58	52.56	169	49.1	9.46	46.93	51.28
25–34	126	51.66	10.76	50.49	52.82	133	51.29	7.84	49.29	53.28	259	51.47	9.63	50.3	52.64
35–44	164	51.84	9.81	50.37	53.31	167	51.27	8.98	49.65	52.89	331	51.55	9.41	50.46	52.65
45–54	137	52.33	10.08	50.85	53.81	135	54.18	8.29	52.89	55.47	272	53.24	9.27	52.26	54.23
55–64	154	54.57	7.54	53.4	55.73	132	53.91	7.79	52.64	55.19	286	54.27	7.66	53.41	55.12
65 +	239	54.36	5.97	53.19	55.53	144	53.89	9.35	52.76	55.02	383	54.18	7.32	53.33	55.02
Total	902	52.82	8.82	52.27	53.37	798	52.38	8.90	51.64	53.12	1700	52.61	8.88	52.16	53.07
Pain intensity (0–10 NRS)															
18–24	82	2.65	3.11	2.17	3.14	87	1.21	0.95	0.68	1.74	169	1.91	2.11	1.55	2.27
25–34	126	2.67	3.29	2.32	3.03	133	1.79	1.77	1.34	2.24	259	2.22	2.57	1.93	2.51
35–44	164	2.62	2.52	2.24	3	167	1.99	1.96	1.64	2.35	331	2.3	2.26	2.04	2.56
45–54	137	3.01	2.86	2.59	3.43	135	2.13	2.42	1.75	2.51	272	2.57	2.69	2.29	2.86
55–64	154	2.69	2.70	2.27	3.11	132	2.37	2.55	1.95	2.79	286	2.54	2.64	2.25	2.84
65 +	239	3.34	2.20	2.91	3.77	144	2.11	2.43	1.81	2.4	383	2.88	2.47	2.59	3.17
Total	902	2.89	2.73	2.72	3.07	798	1.98	2.12	1.82	2.14	1700	2.46	2.49	2.34	2.58

For PROMIS-29+2 scales of function (i.e. physical function, social roles and cognitive function) a higher score corresponds to a better HRQoL and for symptoms (i.e. anxiety, depression, fatigue, sleep disturbance and pain interference) a higher score corresponds to worse HRQoL. Higher score on pain intensity NRS indicate worst pain

*HRQoL* health-related quality of life, *NRS* numeric rating scale

**Fig. 2:**
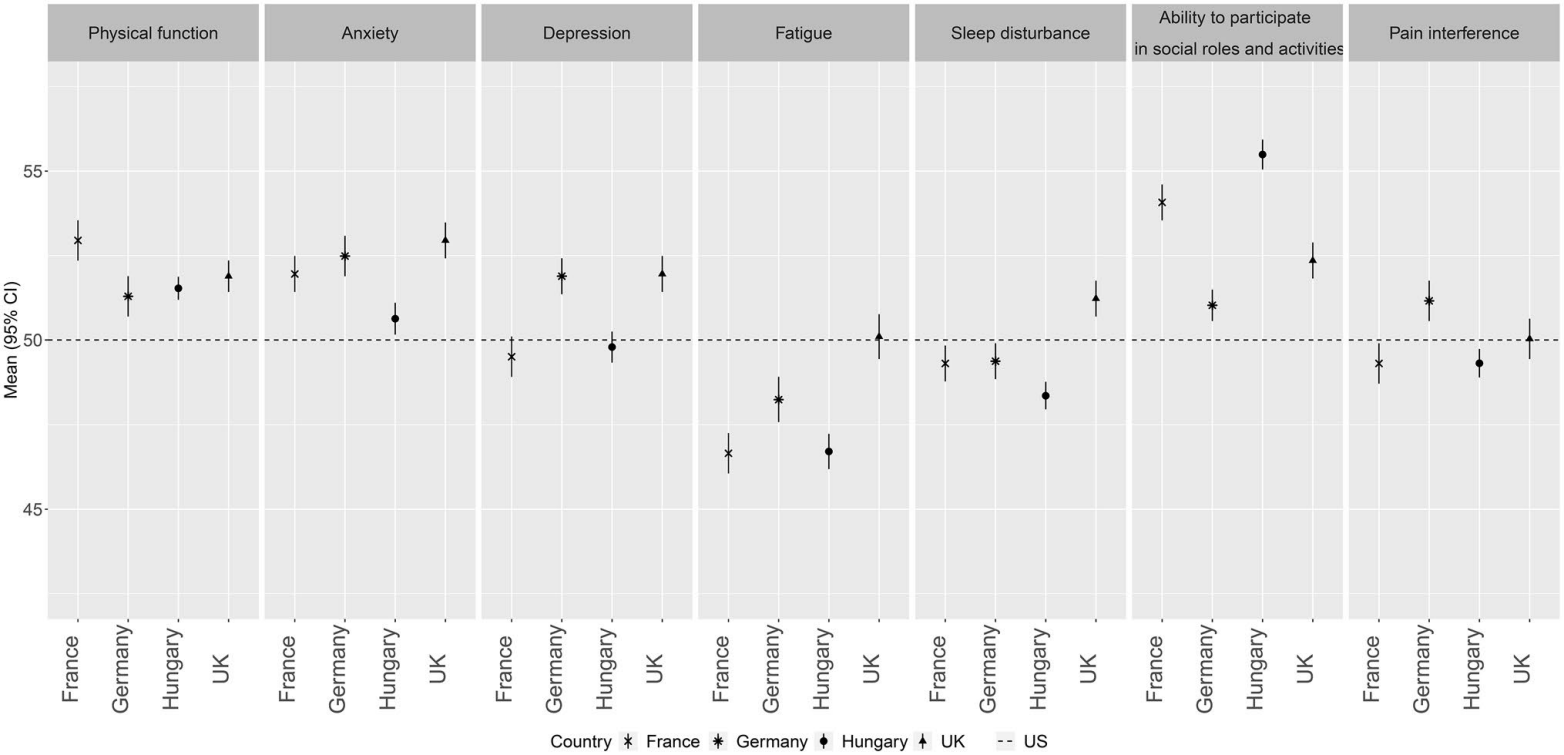
Comparison of domain T-scores in the general population across Hungary, the US, France, Germany and the UK. Note that the cognitive function domain is not presented in the figure due to the lack of data from general population samples in any of the Western European countries. For PROMIS-29+2 scales of function (i.e., physical function, social roles and cognitive function) a higher score corresponds to a better HRQoL and for symptoms (i.e., anxiety, depression, fatigue, sleep disturbance and pain interference) a higher score corresponds to worse HRQoL. *HRQoL* health-related quality of life

Compared to the US calibration sample with a mean of 50 and the three European countries with existing reference values, mean PROMIS-29+2 domain T-scores in the Hungarian general population indicated similar or better HRQoL with the largest difference being seen for social roles (> 5 points from the US calibration sample) (Fig. 2). The lowest level of anxiety and sleep disturbance was found in Hungary, while for physical function it was similar to Germany and the UK and for depression, fatigue and pain interference to France. Cognitive function in Hungary was better compared to the US calibration sample.

## Discussion

This study assessed the psychometric properties of the Hungarian version of PROMIS-29+2 and provided reference values in a large representative sample of the adult general population in Hungary. Our findings provide evidence of a satisfactory measurement performance of the Hungarian PROMIS-29+2. Floor and ceiling effects were observed for nearly all domains depending on the scale orientation that is comparable to the findings of previous studies in various patient samples [18, 20, 21, 25]. An acceptable reliability was confirmed for all domains. Favourable psychometric properties of the scale include an excellent convergent validity with SF-36 and no or minor DIF for main sociodemographic characteristics. Nevertheless, few potential weaknesses of PROMIS-29+2 have also been identified, particularly the poor performance of the sleep disturbance domain.

While the GRM produced an acceptable fit for six PROMIS-29+2 domains, sleep disturbance failed to meet any fit indices and showed item misfit for all four items of the domain and very low item discrimination ability. Sleep109 (‘sleep quality’) vs. Sleep20 (‘problem with sleep’) showed local dependence suggesting redundancy between the two items. Furthermore, response categories of item Sleep116 (‘refreshing sleep’) were disordered and its discriminatory ability was also substantially lower than that of any other item. Similarly to our findings, the Norwegian and Dutch PROMIS-29 validation studies also reported problems with the performance of the sleep disturbance domain and item characteristics curves of Sleep116 [27, 30]. The sleep disturbance domain of PROMIS-29 is unique in the sense that it includes two positively phrased, reverse coded items (Sleep109 and Sleep116). In questionnaires, reverse-worded items are typically intended to reduce response bias (e.g., pattern answering), disrupt nonsubstantive responding or provide a better coverage of the domain studied [55]. Yet, several studies reported that such items can lead to measurement problems, including low reliability and poor model fit and some argue that they would prevent respondents from inattentive or acquiescent answering [56]. The further exploration of the issues with the sleep disturbance domain as well as testing alternative combinations of items could be subject of future research that administer the full PROMIS sleep item bank.

HRQoL decreased with age for physical and social health domains, but not for the cognitive or mental ones. This finding corresponds to the general population reference values in neighbouring Slovenia that reported worse mental health among young adult respondents using the EQ-5D-5L [57] and to the European reference values for the European Organisation for Research and Treatment of Cancer (EORTC) CAT Core that reported an improving trend for cognitive and emotional functions with age [58]. The better HRQoL of the Hungarian population in some domains compared to Western Europe is an unexpected finding as the average health status in Hungary was found to be below the EU average [59]. Comparisons across countries using different health status measures also reported mixed evidence. Using the EQ-5D-3L, the Hungarian general population was in a substantially worse HRQoL compared to other European countries [60]; however, the EQ-5D lacks domains for fatigue, sleep problems and social roles. By contrast, the EORTC CAT showed that in some HRQoL domains (e.g., physical functioning, social functioning, sleep problems), the Hungarian population, in fact, had a better health status than what was found in Germany or the UK [58].

In this study, we used the official US item parameters to compute T-scores. However, multiple approaches exist to score PROMIS items with each offering their own advantages and disadvantages [61]. Using the US item calibrations follows the PROMIS convention and has the advantage that it represents a common metric, which directly allows for international comparisons. On the other hand, if any item within a domain shows language-DIF, the parameter estimates may not be valid for the local population. Another option is using country-specific item calibrations that enable improved accuracy for comparisons with local patient groups and country-specific interpretation of scores. To benefit from the advantages of both methods, a hybrid approach may also be recommended that uses US item calibrations for items without language-DIF and country-specific item parameters for items with language-DIF [62].

There are a number of limitations to this study. First, the online mode of administration might be responsible for selection bias, and the quota sampling lacks known sampling probability. Second, data were collected during the second wave of the COVID-19 pandemic in Hungary that could have an effect on self-reported health, particularly on young adults’ mental health [62–67]. However, responses on self-perceived health status (SF-36 first question) were roughly identical to those reported in a similar large-scale general population survey in Hungary before the pandemic (2019) [68]. The third limitation is that we had no information on the total number of potential respondents contacted by the survey company or access to the data from partially completed questionnaires. Fourth, the reference values for the 65 + age group might not be fully representative to the general population as there were relatively few respondents in the 75 + age group (3.4%). Fifth, it was not possible to fit a GRM for cognitive function because the domain has only two items in PROMIS-29+2. Finally, for each PROMIS-29 domain we fitted a GRM, as this modelling approach was used to develop the PROMIS item banks and this is suggested in the PROMIS analytical recommendations [6]. However, it is possible that certain traits measured by PROMIS-29+2 domains do not have an a priori normal distribution in the population, e.g., physical functioning, pain, fatigue, anxiety and depression because many respondents reporting no problems [69]. A few alternative model types exist that could be useful for future analyses, for example, to alleviate the skewness in data, e.g., zero-inflated mixture IRT models or Davidian Curve IRT [70, 71].

In summary, our results provide support for the satisfactory psychometric properties of the Hungarian version of PROMIS-29+2, including internal consistency reliability, good convergent validity with SF-36 and no DIF. However, the large ceiling and floor effect may detract from the usefulness of the measure when the aim is to differentiate between HRQoL levels at the mild end of the scale. Measurement problems were found with regard to the sleep disturbance domain that would require further refinement. Age and gender-specific reference values were generated for the Hungarian PROMIS-29+2 that facilitate the interpretation of HRQoL outcomes in various patient populations.

## Supplementary Information

Below is the link to the electronic supplementary material.

Supplementary file1 (PDF 683 KB)

## Data Availability

are available from the corresponding author upon a reasonable request.
