# Supplementary material for: Hungarian PROMIS-29+2: psychometric properties and population reference values
Source: Qual Life Res. 2023 Feb 15;32(8):2179–94. doi: 10.1007/s11136-023-03364-7 (PMC9931172; doi:10.1007/s11136-023-03364-7)
Supplement: Supplementary file 1 — Supplementary file1 (PDF 683 KB) [file 11136_2023_3364_MOESM1_ESM.pdf]

Supplementary Information

Hungarian PROMIS-29+2: psychometric properties and population reference values

Balázs Jenei, Alex Bató, Ariel Z. Mitev, Valentin Brodszky, Fanni Rencz

Correspondence:
Fanni Rencz
Department of Health Policy
Corvinus University of Budapest
Address: 8 Fővám tér, H-1093 Budapest, Hungary
E-mail: fanni.rencz@uni-corvinus.hu

Online Resource 1 Chen and Thissen's indices (above the diagonal) and local dependence matrix (below the diagonal)

Online Resource 2 Item mean scores conditional on total score minus item score (monotonicity)

Online Resource 3 Item characteristic curves for six PROMIS-29+2 domains

Online Resource 4 Age DTF of the physical function domain of PROMIS-29+2

Online Resource 5 Spearman's correlation matrix between PROMIS-29+2 domains and pain intensity scale

Online Resource 6 Spearman's correlation matrix between SF-36 domains

Online Resource 1 Chen and Thissen's indices (above the diagonal) and local dependence matrix (below the diagonal)

**Physical function**	**PFA11**	**PFA21**	**PFA23**	**PFA53**
PFA11	-	0.062	-0.079	0.091
PFA21	25.92	-	0.071	-0.07
PFA23	42.66	34.747	-	0.058
PFA53	56.643	32.973	23.086	-
**Anxiety**	**EDANX01**	**EDANX40**	**EDANX41**	**EDANX53**
EDANX01	-	0.051	-0.073	-0.091
EDANX40	17.503	-	-0.103	-0.087
EDANX41	36.441	71.46	-	0.134
EDANX53	56.253	51.981	122.212	-
**Depression**	**EDDEP04**	**EDDEP06**	**EDDEP29**	**EDDEP41**
EDDEP04	-	-0.1	-0.076	-0.144
EDDEP06	68.008	-	-0.103	-0.192
EDDEP29	39.193	72.358	-	0.121
EDDEP41	141.661	251.199	99.227	-
**Fatigue**	**HI7**	**AN3**	**FATEXP41**	**FATEXP40**
HI7	-	-0.117	-0.199	0.29
AN3	93.873	-	0.144	-0.133
FATEXP41	270.146	140.85	-	-0.249
FATEXP40	573.351	120.011	420.612	-
**Sleep disturbance**	**Sleep109**	**Sleep116**	**Sleep20**	**Sleep44**
Sleep109	-	0.176	-1.01	-0.331
Sleep116	210.77	-	-0.442	-0.277
Sleep20	6942.895	1329.998	-	0.13
Sleep44	744.461	523.222	115.158	-
**Social roles**	**SRPPER11_CaPS**	**SRPPER18_CaPS**	**SRPPER23_CaPS**	**SRPPER46_CaPS**
SRPPER11_CaPS	-	-0.159	-0.082	-0.211
SRPPER18_CaPS	170.985	-	-0.109	-0.172
SRPPER23_CaPS	45.251	80.85	-	0.171
SRPPER46_CaPS	303.114	201.903	198.827	-
**Pain interference**	**PAININ9**	**PAININ22**	**PAININ31**	**PAININ34**
PAININ9	-	-1.386	-0.121	-0.578
PAININ22	13064.97	-	-0.461	-0.595
PAININ31	99.23	1445.764	-	0.224
PAININ34	2273.466	2405.994	340.236	-

Online Resource 2 Item mean scores conditional on total score minus item score (monotonicity)

Physical Function

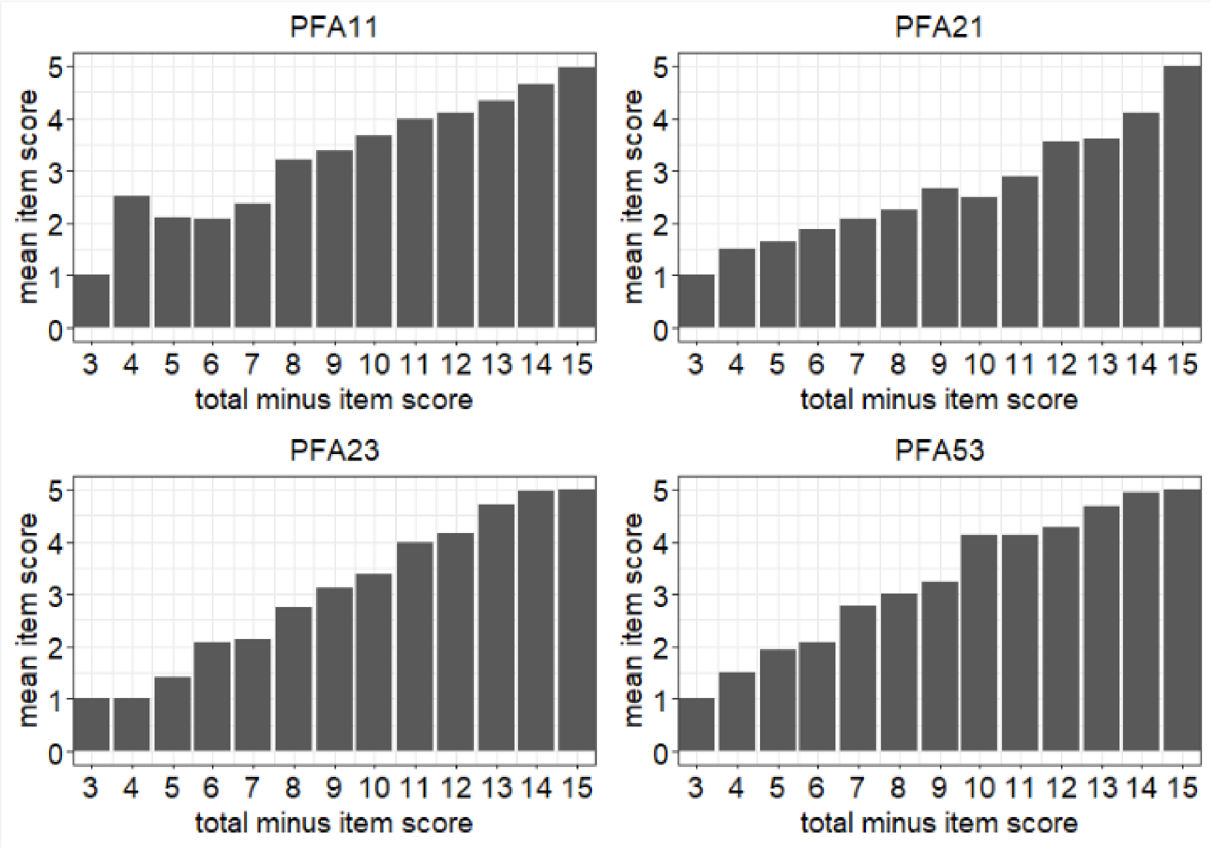

Anxiety

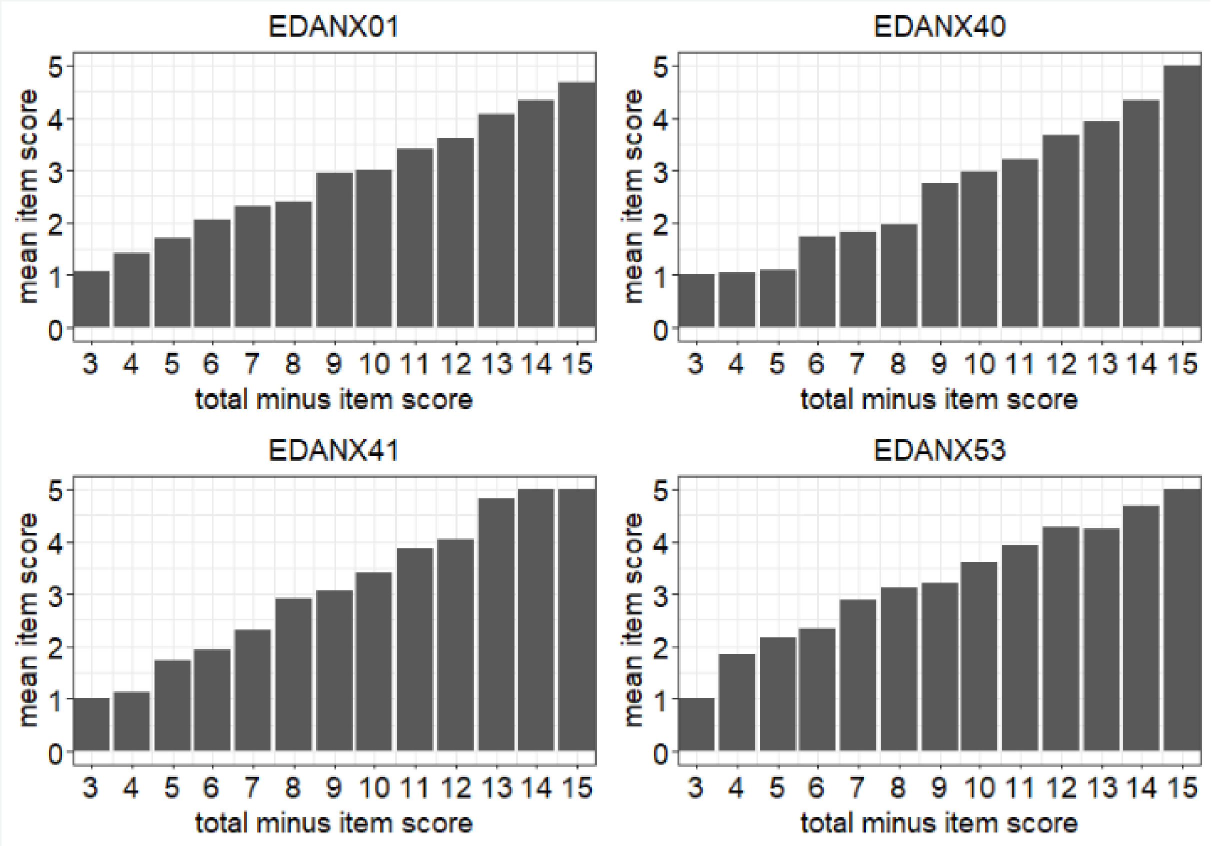

Depression

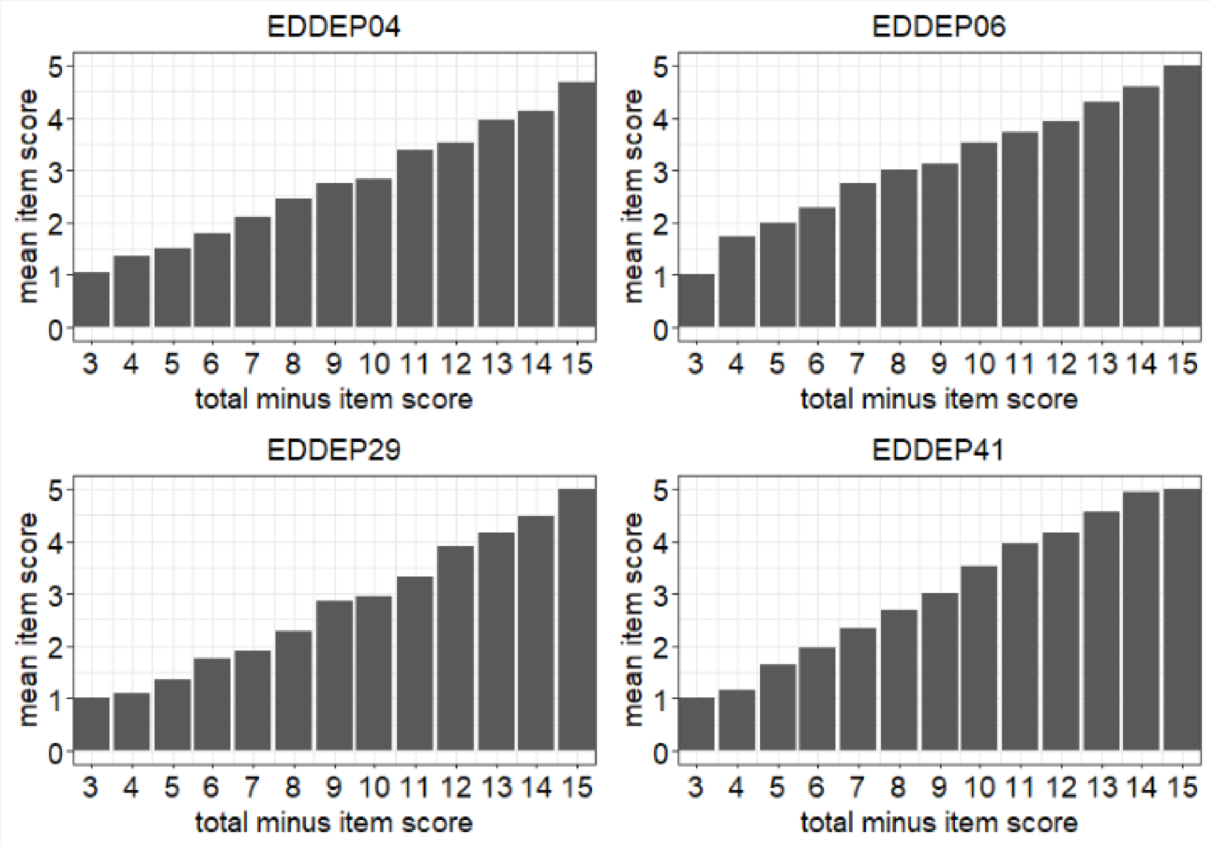

Fatigue

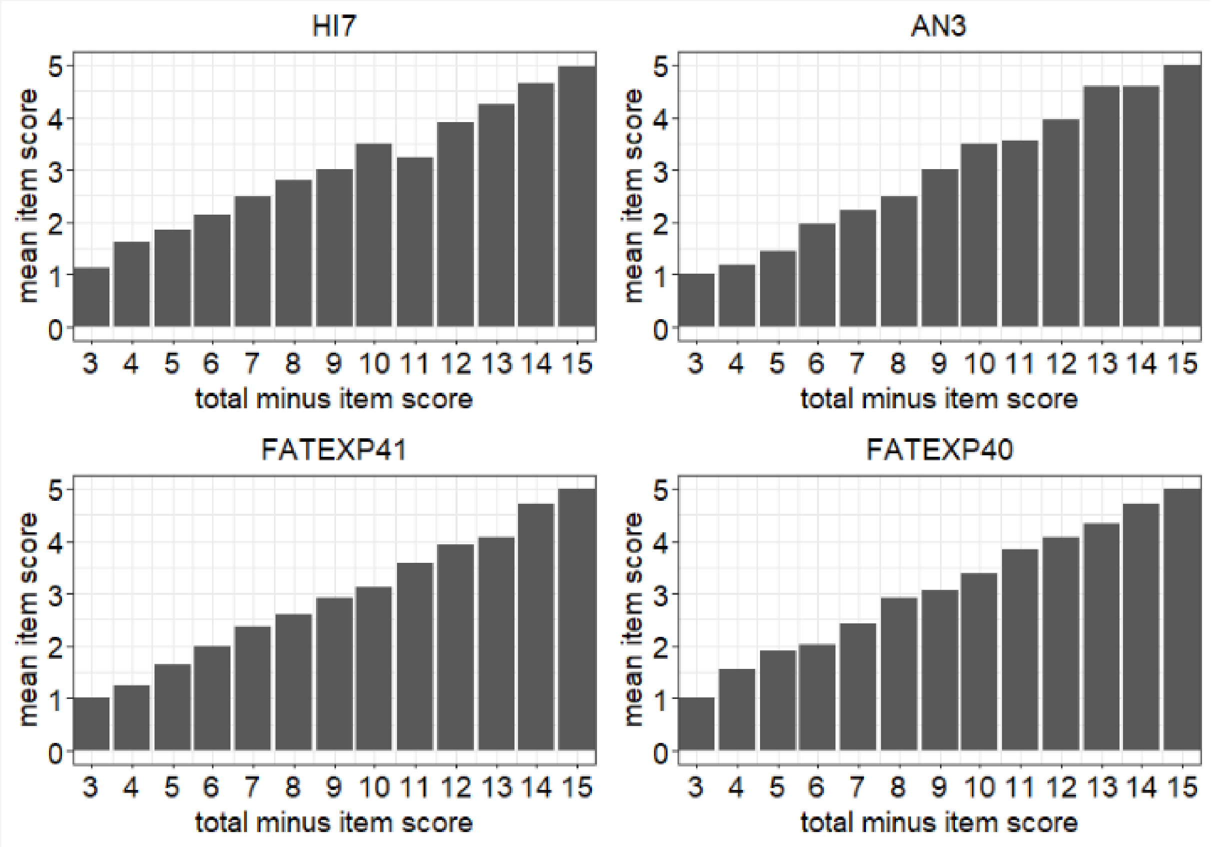

Sleep disturbance

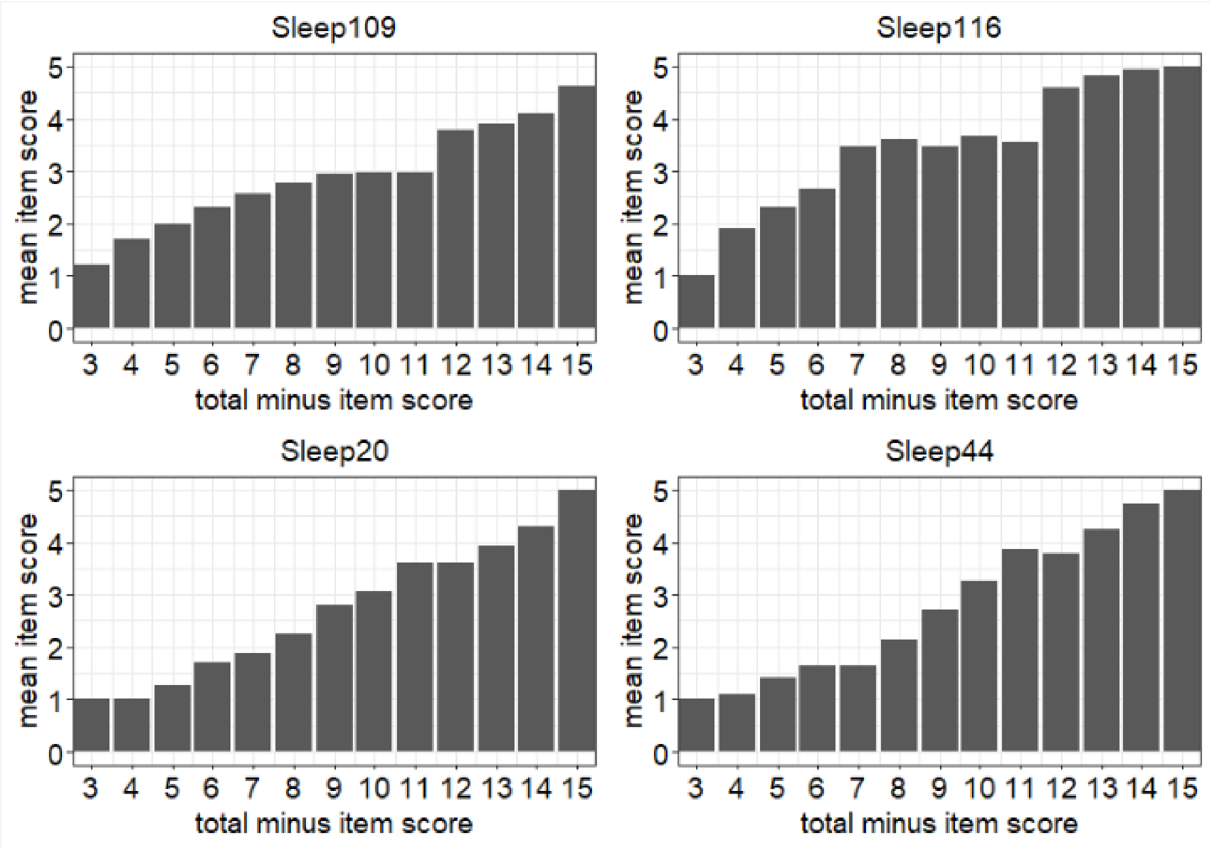

Social Roles

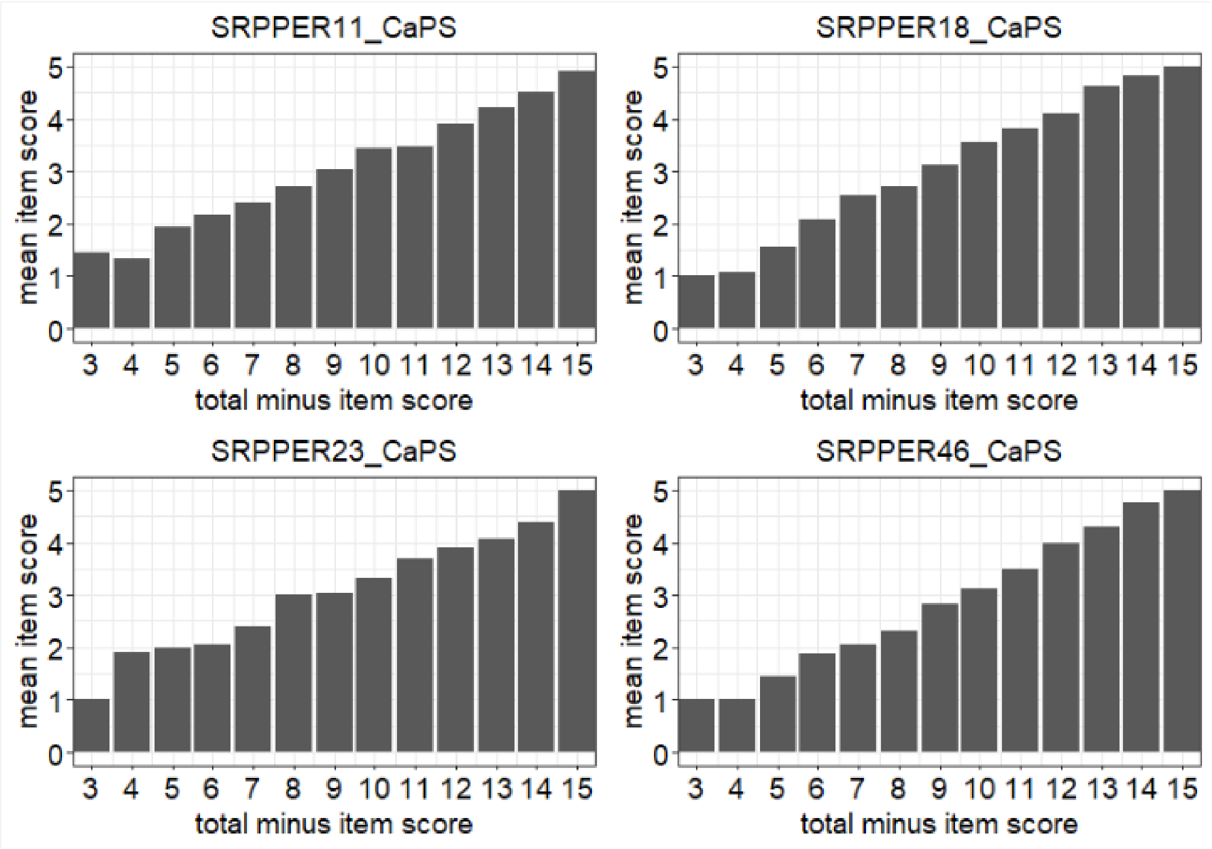

Pain Interference

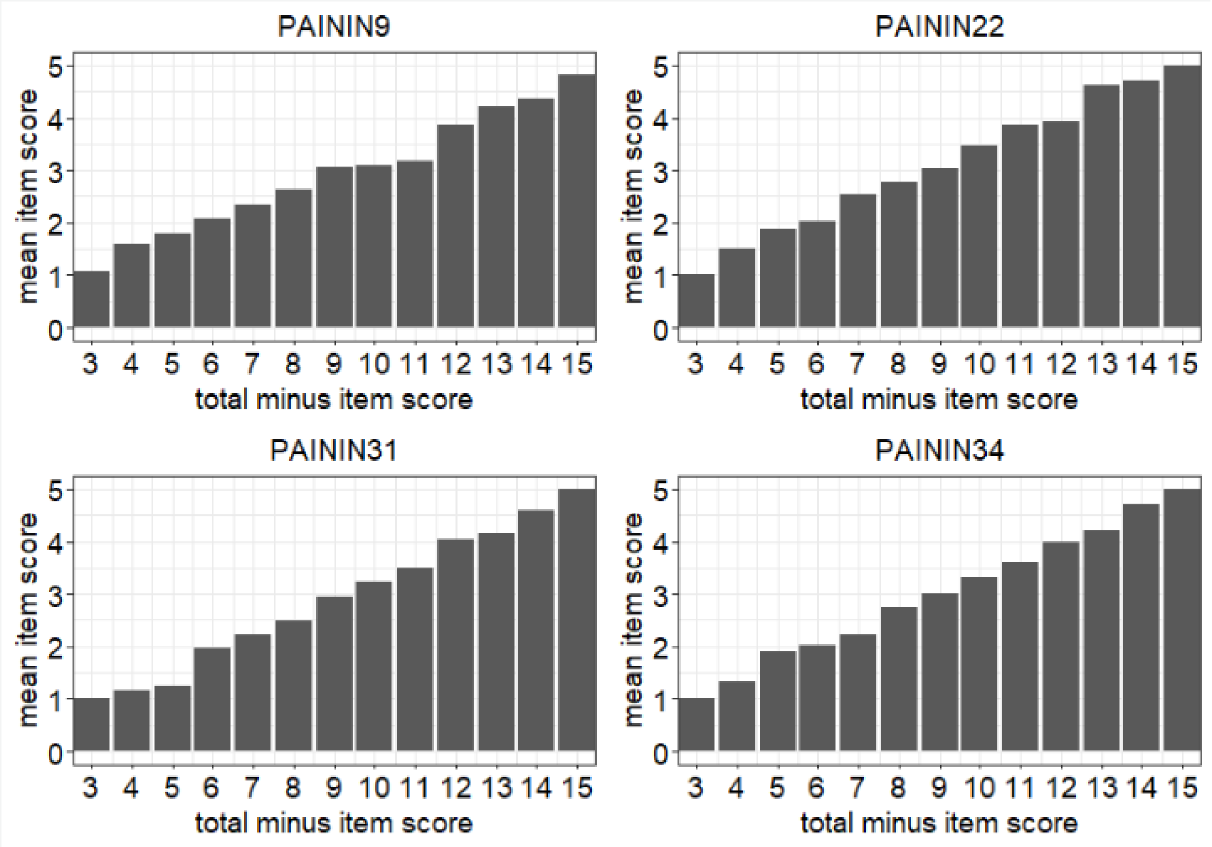

Online Resource 3 Item characteristic curves for six PROMIS-29+2 domains

Physical function

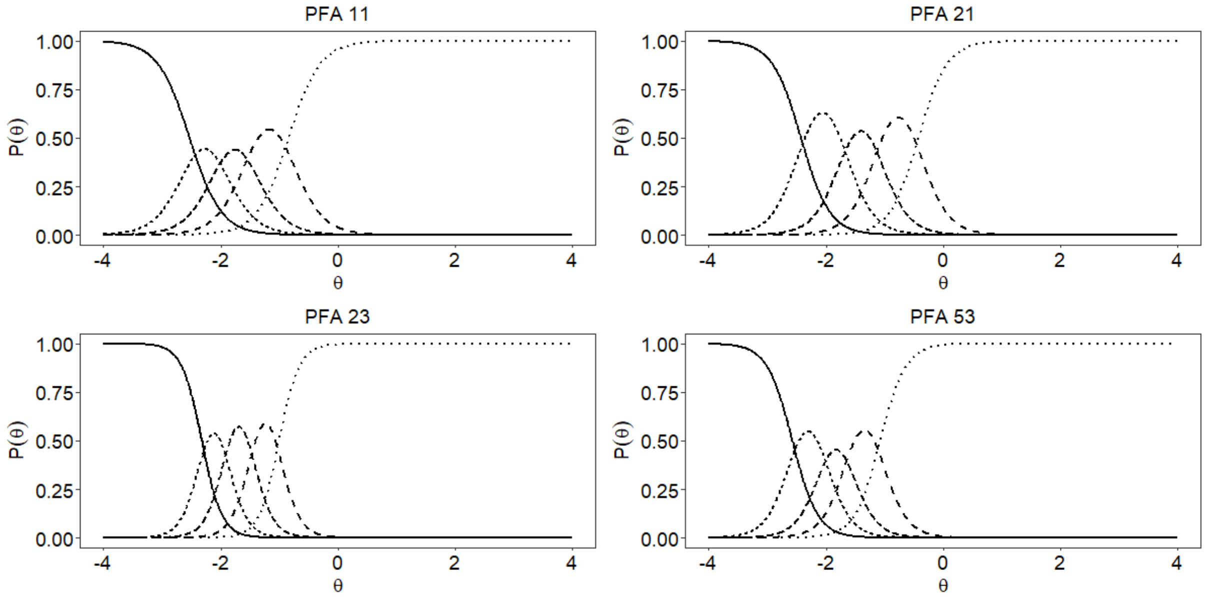

Anxiety

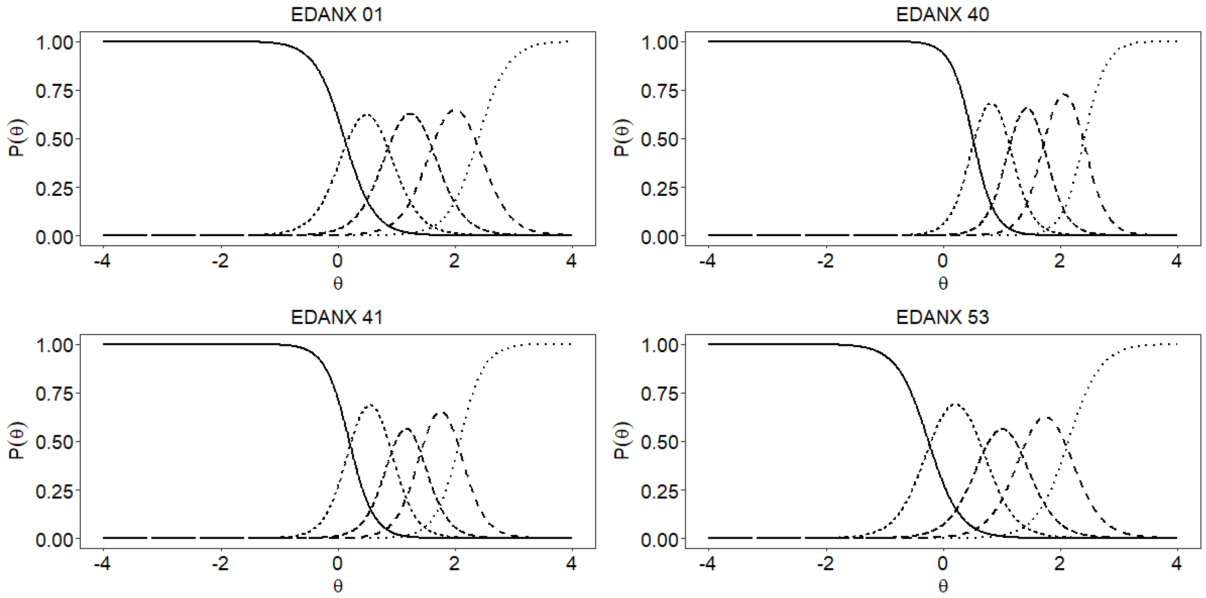

Depression

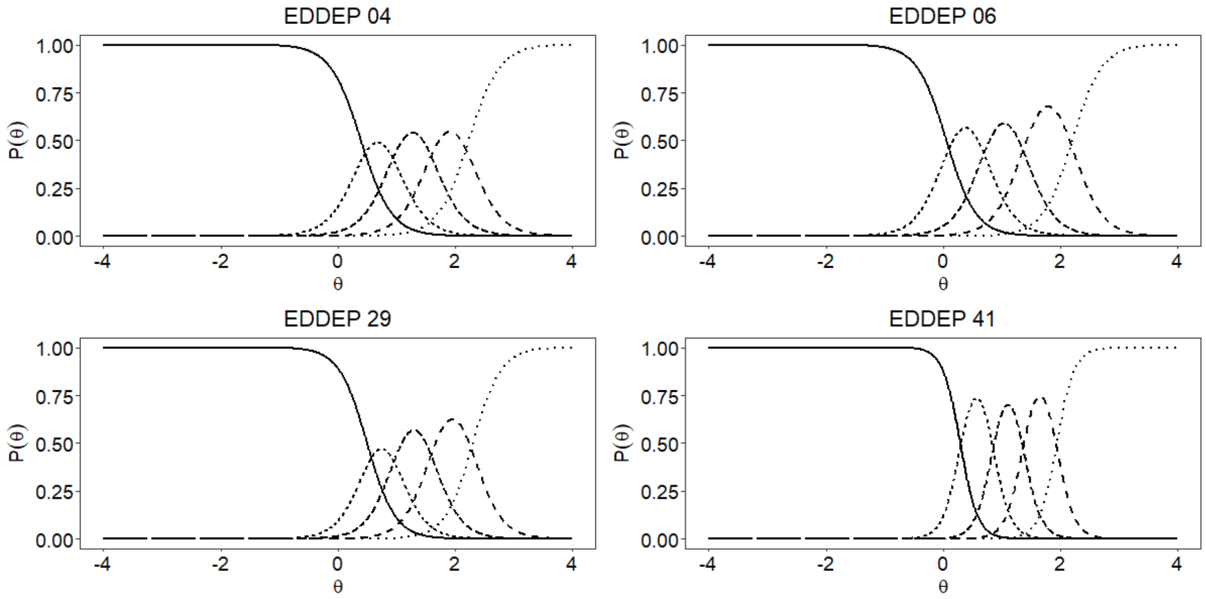

Fatigue

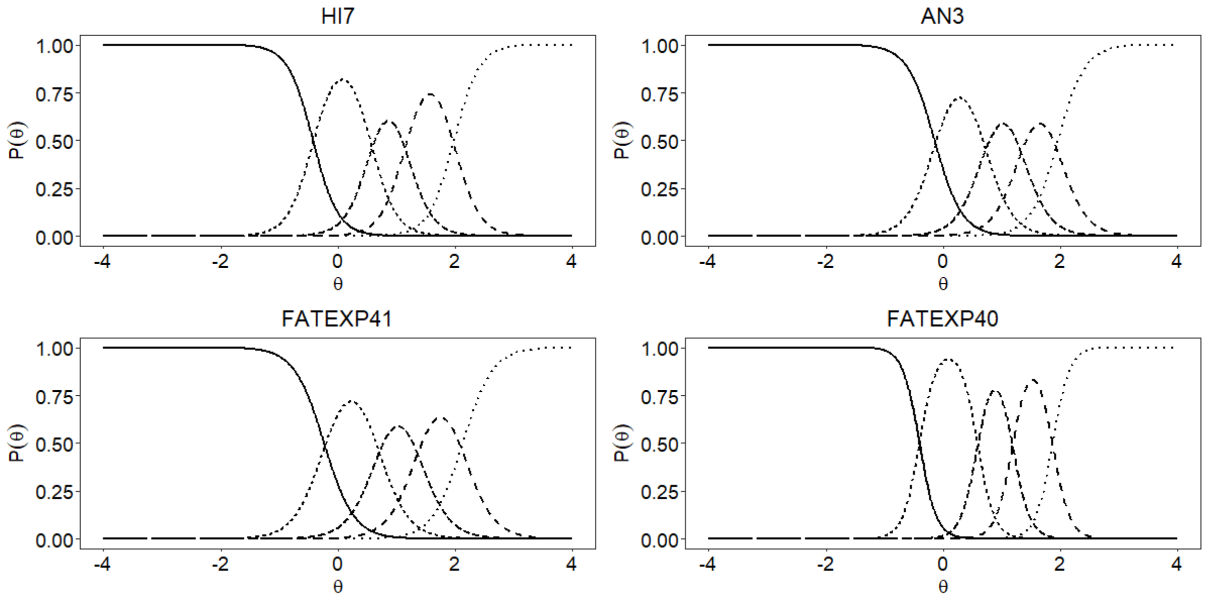

Social roles

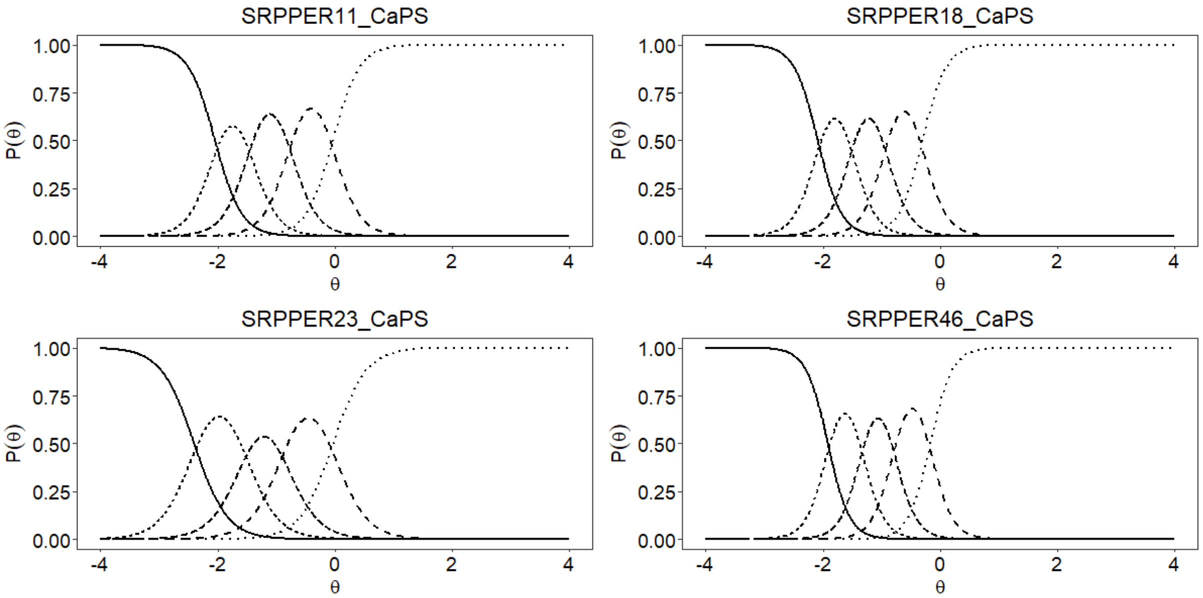

Pain interference

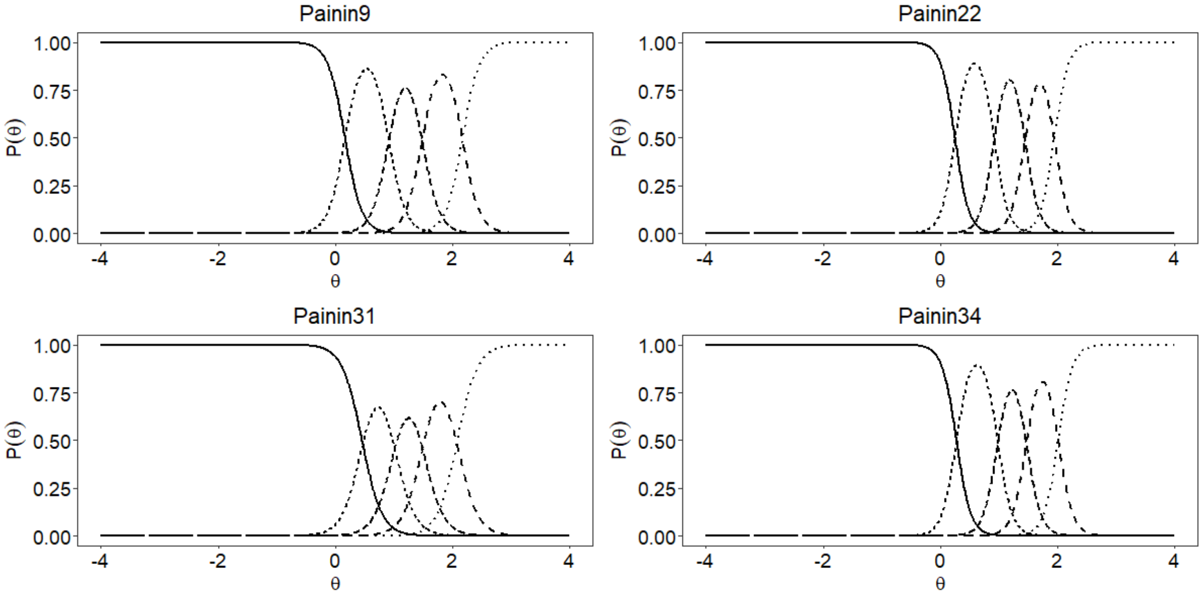

Online Resource 4 Age DTF of the physical function domain of PROMIS-29+2

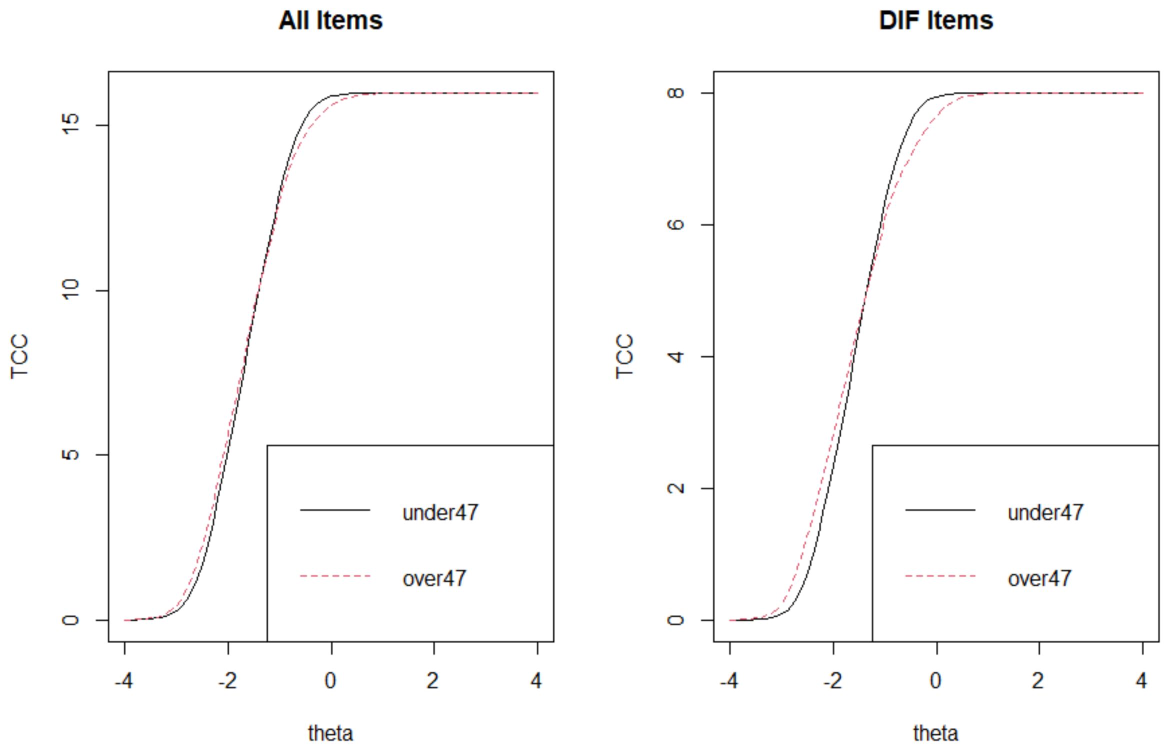

DTF=differential test functioning, TCC = test characteristic curve

Online Resource 5 Spearman's correlation matrix between PROMIS-29+2 domains and pain intensity scale

PROMIS-29+2	Physical function	Anxiety	Depression	Fatigue	Sleep disturbance	Ability to participate in social roles and activities	Pain interference	Cognitive function	Pain intensity (0-10)
**Physical function**	-								
**Anxiety**	-0.28	-							
**Depression**	-0.31	0.78	-						
**Fatigue**	-0.39	0.65	0.64	-					
**Sleep disturbance**	-0.30	0.45	0.44	0.53	-				
**Ability to participate in social roles and activities**	0.55	-0.54	-0.57	-0.61	-0.44	-			
**Pain interference**	-0.60	0.38	0.40	0.50	0.39	-0.61	-		
**Cognitive function**	0.18	-0.33	-0.36	-0.32	-0.33	0.32	-0.25	-	
**Pain intensity (0-10)**	-0.49	0.35	0.36	0.50	0.39	-0.49	0.74	-0.25	-

For PROMIS-29+2 scales of function (i.e. physical function, social roles and cognitive function) a higher score corresponds to a better HRQoL and for symptoms (i.e. anxiety, depression, fatigue, sleep disturbance and pain interference) a higher score corresponds to worse HRQoL. HRQoL = health-related quality of life
$p<0.05$ for all correlation coefficients.

Online Resource 6 Spearman's correlation matrix between SF-36 domains

SF-36	PF	RP	RE	VT	MH	SF	BP	GH
**PF**	-							
**RP**	0.65	-						
**RE**	0.42	0.62	-					
**VT**	0.39	0.46	0.50	-				
**MH**	0.29	0.35	0.46	0.81	-			
**SF**	0.39	0.47	0.51	0.59	0.62	-		
**BP**	0.61	0.62	0.46	0.53	0.43	0.51	-	
**GH**	0.63	0.52	0.42	0.56	0.48	0.47	0.56	-

BP=bodily pain; GH=general health; MH=mental health; PF = physical functioning; RE=role limitations due to emotional problems; RP=role limitations due to physical health; SF=social functioning; VT=vitality
$p<0.05$ for all correlation coefficients.
